# A Novel Two-Step Channel Estimation Method for RIS-Assisted mmWave Systems

**DOI:** 10.3390/s24165362

**Published:** 2024-08-19

**Authors:** Jiarun Yu

**Affiliations:** School of Information and Communications Engineering, Beijing University of Posts and Telecommunications, Beijing 100876, China; yujiarun@bupt.edu.cn

**Keywords:** channel estimation, RIS-assisted mmWave systems, MMPGA, ResNet, multi-task regression model, MTRnet

## Abstract

In this work, we resolve the cascaded channel estimation problem and the reflected channel estimation problem for the reconfigurable intelligent surface (RIS)-assisted millimeter-wave (mmWave) systems. The novel two-step method contains modified multiple population genetic algorithm (MMPGA), least squares (LS), residual network (ResNet), and multi-task regression model. In the first step, the proposed MMPGA-LS optimizes the crossover strategy and mutation strategy. Besides, the ResNet achieves cascaded channel estimation by learning the relationship between the cascaded channel obtained by the MMPGA-LS and the channel of the user (UE)-RIS-base station (BS). Then, the proposed multi-task-ResNet (MTRnet) is introduced for the reflected channel estimation. Relying on the output of ResNet, the MTRnet with multiple output layers estimates the coefficients of reflected channels and reconstructs the channel of UE-RIS and RIS-BS. Remarkably, the proposed MTRnet is capable of using a lower optimization model to estimate multiple reflected channels compared with the classical neural network with the single output layer. A series of experimental results validate the superiority of the proposed method in terms of a lower norm mean square error (NMSE). Besides, the proposed method also obtains a low NMSE in the RIS with the formulation of the uniform planar array.

## 1. Introduction

Intelligent reflecting surfaces (IRS), also denoted as reconfigurable intelligent surfaces (RISs), have the potential to improve the system performance of the 6G network [1]. Generally speaking, RIS was one kind of meta-surface composed of a vast number of passive reflecting elements, which could be controlled in real time to dynamically alter the amplitude and/or phase of the reflecting signal, thus collaboratively enabling smart reconfiguration of the radio propagation environment [2]. However, channel estimation in passive RIS-assisted millimeter-wave (mmWave) systems is challenging. It is because the passive RIS is unable to perform signal processing, and the large number of reflecting elements in the RIS leads to high complexity [3,4].

During the past decade, some methodologies have been used to address channel estimation. The authors of [5] proposed a tensor modeling approach aiming at reducing the channel estimation error. This channel estimation problem was translated into recovering multiple rand-1 matrix approximation sub-problems [5]. Authors of [6] investigated the direction-of-departure (DoD) and direction-of-arrival (DoA) estimation in a bistatic multiple input multiple output (MIMO) radar, in which a backward IRS was exploited to receive the echoes reflected by the targets from the NLOS viewpoint. Wei et al. [7] applied the least-squares (LS) channel estimation solution for the channel of the user (UE)-base station (BS). However, the channel estimation performance in [7] was sensitive to the additional Gaussian white noise. Compressed sensing methods in [8,9] transformed the channel estimation into a sparse signal recovery problem. The channel estimation method based on compressed sensing required traversing the dictionary matrix to attain the low norm mean square error (NMSE). In [10], authors developed an improved version of the differential evolution (DE) algorithm for cell-free MIMO systems assisted by RIS. By configuring phase shift vectors for the RIS-assisted reflected channel estimation, Byun et al. [11] improved the channel estimation accuracy. The evolution algorithm used in [11] paid attention to the improvement from the mutation operation and neglected the influence due to a random children selection in the crossover strategy. The convex optimization approach [12] and hybrid evolution method [13] reduced the error between the actual signal and the reconstructed signal via optimizing the corresponding channel matrix.

As a popular approach for improving communication systems performance, some researchers have introduced deep learning for the channel estimation problem [14,15,16]. In [14,15], the authors proposed a convolutional-neural-network (CNN)-based channel estimation method. The method in [14] required the RIS to process the transmitted signal. Therefore, this method could not be applied to the passive RIS system. A conditional generative adversarial network (cGAN) was designed to estimate the cascaded channel with the received signals as conditional information [16]. A deep-learning-based approach with the traditional orthogonal matching pursuit followed by the residual network was introduced for the cascaded uplink channel estimation problem [17]. However, the methods in [16,17] did not achieve the channel estimation of RIS-BS and UE-RIS. Without the information about the directive channel of UE-BS, a deep-learning-based channel estimation method in [18] did not estimate the reflected channels of RIS-BS and UE-RIS effectively.

To further reduce the channel estimation NMSE, some methods integrated deep learning and other methods [19,20,21]. In [19], Wang et al. proposed a channel estimation scheme based on an LS solution for estimating the cascaded channel. Differently, the authors of [20] modeled the channel estimation as a denoising problem and developed a versatile deep residual learning-based cascaded channel estimation framework. Besides, the channel estimation method adopted a CNN-based deep residual network to learn the mapping between the noisy channel matrix and the denoised channel matrix [21]. The optimized network architectures in [19,20,21] did not estimate the reflected channels of RIS-BS and UE-RIS simultaneously. Wang et al. proposed a machine learning-based CS channel estimation method for wireless communication [22]. In [23], authors propose a channel estimation method for the passive RIS-assisted systems. The authors of [24] performed two stages by following atomic norm minimization to recover the channel parameters. In [25], authors proposed a strategy for joint target and user assignment, power allocation, and subchannel allocation (JCAPASA) in the RIS-assisted systems. The framework used in [26] integrated the CNN and Lagrange optimization algorithms, which aimed at achieving cascaded channel estimation. The method in [26] required the additional optimization of Lagrange factors to obtain the low channel estimation NMSE.

Most of the above-mentioned methods mainly focused on cascaded channel estimation and did not simultaneously achieve the channel estimation of RIS-BS and UE-RIS without considering the UE-to-BS communication. To overcome this shortcoming, we propose a novel two-step channel estimation method for the RIS-assisted mmWave systems. The scope of this work is to fill in the gap in the literature on reflected channel estimation with the use of deep learning. The residual network (ResNet) with the cross-layers operation [27] further improves the non-linear processing ability relative to some common neural networks. Compared with the single regression model, the multi-task model [28] has stronger learning ability. With the multiple output layers, the multi-task solved many sub-problems simultaneously. Therefore, we introduce a neural network integrating the multi-task regression model and ResNet for the channel estimation problem. Remarkably, the two-step method integrates the proposed modified multiple population genetic algorithm (MMPGA), LS estimator, ResNet, and multi-task-ResNet (MTRnet). The main contributions of this paper are summarized as follows:

- In addition to the cascaded channel estimation, we further estimate the reflected channels of RIS-BS and UE-RIS. Remarkably, a novel two-step channel estimation method using MMPGA, LS estimator, ResNet, and MTRnet is introduced for the RIS-assisted mmWave systems.
- The MMPGA-LS-ResNet is proposed to estimate the cascaded channel of UE-RIS-BS. The MMPGA-LS optimizes the crossover strategy and mutation strategy compared with the common evolution algorithm. As a result, the proposed MMPGA-LS is capable of reducing the estimation error. Then, ResNet is applied to further reduce the cascaded channel error. Relying on the designed network architecture, including the multiple cross-layer operations and layers, the proposed ResNet learns the relationship between the output of MMPGA-LS and the channel of UE-RIS-BS effectively.
- Furthermore, the proposed MTRnet is introduced for estimating the reflected channels of RIS-UE and UE-RIS. Compared with the single regression model, the MTRnet integrates the multi-task learning model and ResNet. As a result, the proposed MTRnet with multiple output layers achieves the reflected channel estimation within fewer optimization models compared with that based on the single regression model.
- A series of experimental results have validated the superiority of the novel two-step channel estimation method. For the cascaded channel estimation performance, the MMPGA-LS achieves a lower NMSE compared with a genetic algorithm (GA) [29] and particle swarm algorithm (PSO) [30]. Besides, the proposed ResNet also obtains a lower NMSE compared with convolutional recurrent neural network (CRNN) [17] and CNN [15]. Additionally, the proposed MTRnet based on the multi-task learning ability still outperforms some single-learning models in terms of a lower NMSE. Besides, the proposed method also obtains a low NMSE in the RIS with the formulation of the uniform planar array.

The rest of this paper is organized into the following parts: In Section 2, the problem of channel estimation is introduced. In Section 3, the proposed MMPGA-ResNet-MTRnet-based method is described in detail. In Section 4, the proposed two-step method is utilized for the simulations of channel estimation. The numerical results compared with other algorithms are presented to validate the effectiveness of the proposed method. The conclusions are given in Section 5.

## 2. Channel Estimation System Model

In the uplink RIS-assisted mmWave communication systems, there is no point-to-point communication from UE to BS. Considering the *N*-elements BS with the formulation of a uniform linear array and *M*-elements RIS with the formulation of a uniform linear array [31], the received signal at the BS is given [32,33]

(1) $$y=H_{1}\Psi H_{2}s+n,$$

where $H_{1}\in C^{N\times M}$ denotes the channel of RIS-BS, $\Psi =\mathrm{diag}\left[b_{1}e^{j\psi_{1}},b_{2}e^{j\psi_{2}},\cdots ,b_{M}e^{j\psi_{M}}\right]\in C^{M\times M}$ represents the reflecting matrix, and $\psi_{m}$ is distributed in the interval [−π/2,+π/2]. The channel of UE-RIS is denoted as $H_{2}\in C^{M\times 1}$, the transmitted pilot signal sequence with the length $L_{s}$ is s [34], the Gaussian white noise with mean 0 and variance $\xi_{n}^{2}$ is n.

In the RIS with the formulation of ULA, $H_{\mathrm{ULA},1}$ is expressed as

(2) $$H_{\mathrm{ULA},1}=\sqrt{\frac{NM}{P}}\sum_{p=1}^{P}\alpha_{1,p}a_{\mathrm{BS},\mathrm{Rx}}\left(\theta_{p}\right)a_{\mathrm{RIS},\mathrm{Tx}}^{H}\left(\omega_{p}\right),$$

where the number of multipaths is *P*, $\alpha_{1,p}$ is the complex gain, $a_{\mathrm{BS},\mathrm{Rx}}\left(\theta_{p}\right)C^{N\times 1}$ denotes the steering vector at the BS side, $a_{\mathrm{RIS},\mathrm{Tx}}\left(\omega_{p}\right)C^{M\times 1}$ represents the steering vector from the departure direction at the RIS side, $\theta_{p}$ means the physical direction-of-arrival (DoA) at the BS side, $\omega_{p}$ is the direction-of-departure (DoD) at the RIS side, and ${\left(\cdot \right)}^{H}$ expresses the conjugate transport operation, $\alpha_{1}={\left\{\alpha_{1,p}\right\}}_{p=1,\cdots ,P}$. The multipaths in the systems contain the single line-of-sight (LOS) path and P−1 non-line-of-sight (NLOS) paths.

(3) $$a_{\mathrm{BS},\mathrm{Rx}}\left(\theta_{p}\right)=\frac{1}{\sqrt{N}}{\left[e^{j\frac{2\pi }{\lambda }dn\sin\left(\theta_{p}\right)}\right]}^{T},n=0,\cdots ,N-1,$$

where λ means the wavelength of the barrier frequency, $d=\frac{\lambda }{2}$ is the spacing between adjacent elements, and ${\left(\cdot \right)}^{T}$ presents the transport operation. The steering vector $a_{\mathrm{RIS},\mathrm{Tx}}\left(\omega_{p}\right)$ is expressed as

(4) $$a_{\mathrm{RIS},\mathrm{Tx}}\left(\omega_{p}\right)=\frac{1}{\sqrt{M}}{\left[e^{j\frac{2\pi }{\lambda }dm\sin\left(\omega_{p}\right)}\right]}^{T},m=0,\cdots ,M-1.$$

$H_{\mathrm{ULA},2}$ is given as

(5) $$H_{\mathrm{ULA},2}=\sqrt{\frac{M}{P}}\sum_{p=1}^{P}\alpha_{2,p}a_{\mathrm{RIS},\mathrm{Rx}}\left(\phi_{p}\right),$$

where $\alpha_{2,p}$ is the complex gain; the steering vector at the arrival direction of RIS side is represented as $a_{\mathrm{RIS},\mathrm{Rx}}\left(\phi_{p}\right)$; $\phi_{p}$ is DoA at the RIS side; $\alpha_{2}={\left\{\alpha_{2,p}\right\}}_{p=1,\cdots ,P}$

(6) $$a_{\mathrm{RIS},\mathrm{Rx}}\left(\phi_{p}\right)=\frac{1}{\sqrt{M}}{\left[e^{j\frac{2\pi }{\lambda }dm\sin(\phi_{p)}}\right]}^{T},m=0,\cdots ,M-1.$$

In the RIS with the formulation of UPA, $H_{\mathrm{UPA},1}$ is expressed as

(7) $$H_{\mathrm{UPA},1}=\sqrt{\frac{NM}{P}}\sum_{p=1}^{P}\alpha_{1,p}a_{\mathrm{BS},\mathrm{Rx}}\left(\theta_{p}\right)a_{\mathrm{RIS},\mathrm{Tx}}^{H}\left(\beta_{1,p},\delta_{1,p}\right),$$

(8) $$a_{\mathrm{RIS},\mathrm{Tx}}\left(\beta_{p},\delta_{p}\right)=a_{\mathrm{Tx},x}\left(\beta_{1,p},\delta_{1,p}\right)⊗a_{\mathrm{Tx},y}\left(\beta_{1,p},\delta_{1,p}\right),$$

(9) $$a_{\mathrm{Tx},x}\left(\beta_{1,p},\delta_{1,p}\right)=\frac{1}{\sqrt{M_{x}}}{\left[e^{j\frac{2\pi }{\lambda }dm_{x}\sin\left(\beta_{1,p}\right)\sin\left(\delta_{1,p}\right)}\right]}^{T},m_{x}=0,\cdots ,M_{x}-1,$$

(10) $$a_{\mathrm{Tx},y}\left(\beta_{1,p},\delta_{1,p}\right)=\frac{1}{\sqrt{M_{y}}}{\left[e^{j\frac{2\pi }{\lambda }dm_{y}\sin\left(\beta_{1,p}\right)\cos\left(\delta_{1,p}\right)}\right]}^{T},m_{y}=0,\cdots ,M_{y}-1,$$

where $M=M_{x}M_{y}$. $H_{\mathrm{UPA},2}$ is given as

(11) $$H_{\mathrm{UPA},2}=\sqrt{\frac{M}{P}}\sum_{p=1}^{P}\alpha_{2,p}a_{\mathrm{RIS},\mathrm{Rx}}\left(\beta_{2,p},\delta_{2,p}\right),$$

(12) $$a_{\mathrm{RIS},\mathrm{Rx}}\left(\beta_{p},\delta_{p}\right)=a_{\mathrm{Rx},x}\left(\beta_{2,p},\delta_{2,p}\right)⊗a_{\mathrm{Rx},y}\left(\beta_{2,p},\delta_{2,p}\right),$$

(13) $$a_{\mathrm{Rx},x}\left(\beta_{2,p},\delta_{2,p}\right)=\frac{1}{\sqrt{M_{x}}}{\left[e^{j\frac{2\pi }{\lambda }dm_{x}\sin\left(\beta_{2,p}\right)\sin\left(\delta_{2,p}\right)}\right]}^{T},m_{x}=0,\cdots ,M_{x}-1,$$

(14) $$a_{\mathrm{Rx},y}\left(\beta_{1,p},\delta_{1,p}\right)=\frac{1}{\sqrt{M_{y}}}{\left[e^{j\frac{2\pi }{\lambda }dm_{y}\sin\left(\beta_{2,p}\right)\cos\left(\delta_{2,p}\right)}\right]}^{T},m_{y}=0,\cdots ,M_{y}-1,$$

where $\beta_{1,p},\beta_{2,p}$ stand for the elevation angle, $\delta_{1,p},\delta_{2,p}$ mean the azimuth angle.

In the passive RIS system, we select the reflecting elements randomly, where $b_{m}\in \left\{0,1\right\},$m=0,⋯,M−1. According to [16], the cascaded channel H is given as follows:

(15) $$H=H_{1}\Psi H_{2}.$$

According to Equations (1)–(15), there exists a relationship between $\left(s,H_{1},H_{2}\right)$, and y, which is written as

(16) $$f^{-1}\left(s,y\right)=H.$$

(17) $$f^{-1}\left(H\right)=\left\{\alpha ,\theta ,\omega ,\phi \right\},f\left(\alpha ,\theta ,\omega ,\phi \right)\to \left\{H_{1},H_{2}\right\}.$$

The resolvable problem in this paper is expressed as

(18) $$f^{-1}\left(s,y\right)\to \left\{H_{1},H_{2}\right\}.$$

## 3. The Novel Two-Step Channel Estimation Method

The proposed channel estimation method contains the MMPGA-LS-ResNet-based cascaded channel estimation and the MTRnet-based reflected channel estimation. In the first step, the MMPGA-LS executes the population initialization, classification, crossover, adaptive mutation, and reservation strategies. Relying on the generation of MMPGA-LS, the proposed ResNet further improves cascaded channel estimation performance. Based on the predicted cascaded channel, MTRnet with multiple output layers simultaneously estimates the channel coefficients (DoAs, DoDs, and channel gains) in the second step. As a result, the proposed method reconstructs the reflected channels of RIS-BS and UE-RIS.

### 3.1. MMPGA-LS-ResNet-Based Cascaded Channel Estimation

In this subsection, the MMPGA-LS-ResNet-based cascaded channel estimation method is introduced for the RIS-assisted mmWave systems. The MMPGA-LS, with its improved crossover strategy and mutation strategy, initially estimates the cascaded channel. Then, the proposed ResNet learns the non-linear relationship between the cascaded channel obtained by the MMPGA-LS and the channel of the UE-RIS-BS. The ResNet aims at further reducing the channel estimation NMSE.

**Figure 1:**
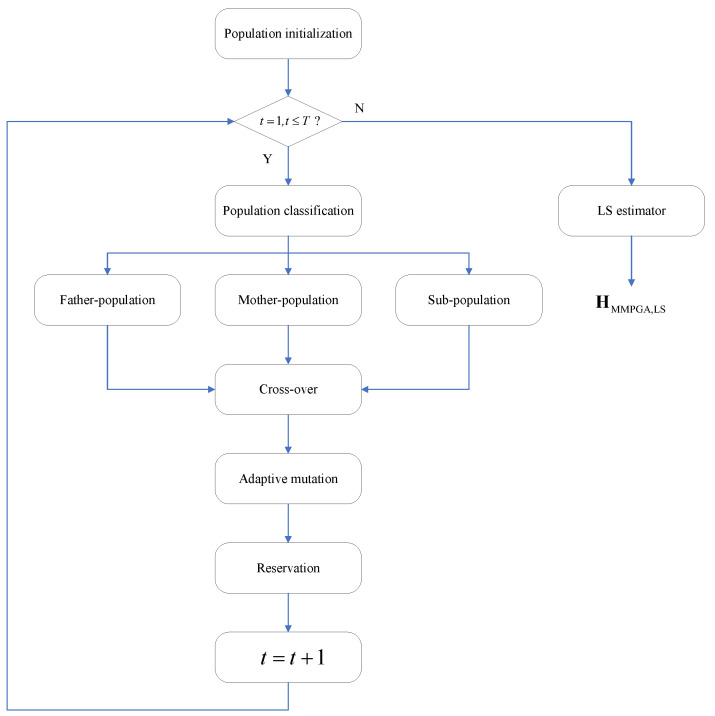
The flowcharts of proposed MMPGA-LS.

The proposed MMPGA-LS reduces the channel estimation error by optimizing the reflecting phases. Based on the population initialization, the MMPGA-LS classifies them via fitness ranking. The MMPGA-LS makes good use of the best one, corresponding to the highest fitness in the crossover strategy. Then, the adaptive mutation strategy flexibly adjusts the mutation factor according to fitness. Based on the generation of the mutation, the proposed method preserves the partial children with higher fitness. After using the LS estimator, the proposed method obtains $H_{\mathrm{MMPGA},\mathrm{LS}}$. Figure 1 represents the flowcharts of the proposed MMPGA-LS.

#### 3.1.1. Population Initialization

We assume that the initial population $\kappa \in R^{Q\times M_{\mathrm{acti}}}$ contains *Q* children, where $M_{\mathrm{acti}}$ denotes the number of active elements in the RIS. κ is given as below

(19) $$\kappa ={\left[\kappa_{1},\kappa_{2},\cdots ,\kappa_{Q}\right]}^{T},$$

where $\kappa_{q}=\left[\kappa_{q,1},\kappa_{q,2},\cdots ,\kappa_{q,M_{\mathrm{acti}}}\right]\in R^{1\times M_{\mathrm{acti}}}$, $\kappa_{q,m_{\mathrm{acti}}}=f_{\mathrm{deci}}(\left\{g_{q,m_{\mathrm{acti}}}\right\}$, $g_{q,m_{\mathrm{acti}}}=\left[g_{q,m_{\mathrm{acti}},1},\cdots ,g_{q,m_{\mathrm{acti}},l},\cdots ,g_{q,m_{\mathrm{acti}},L_{c}}\right]\in Z^{1\times L_{c}}$, $g_{q,m_{\mathrm{acti}},l}\in \left\{0,1\right\}$, $L_{c}$ stands for the length of a binary-gene sequence, and $f_{\mathrm{deci}}\left(\cdot \right)$ means a decimal-transportation function.

(20) $$f_{\mathrm{deci}}\left(g_{q,m_{\mathrm{acti}}}\right)=lb+\frac{\sum_{l=1}^{L_{c}}2^{g_{q,m_{\mathrm{acti}},l}}}{2^{L_{c}}}\left(up-lb\right),l=1,\cdots ,L_{c},$$

where lb is the lower bound, up means the upper bound, $lb\leq \kappa_{q,m_{\mathrm{acti}}}\leq up$. The whole gene population is defined as $G_{\mathrm{init}}\in Z^{Q\times L_{c}\times M_{\mathrm{acti}}}$. $g_{q,m_{\mathrm{acti}},l}$ selects 0 or 1 randomly. 

#### 3.1.2. Population Classification

After using $\kappa_{q}$ to obtain $f_{\mathrm{LS},\mathrm{MMSE}}\left(H_{\mathrm{LS}},H\right)$, the corresponding fitness is defined as

(21) $$fit=\frac{1}{f_{\mathrm{LS},\mathrm{NMSE}}\left(H_{\mathrm{LS}},H\right)},$$

(22) $$f_{\mathrm{LS},\mathrm{NMSE}}\left(H_{\mathrm{LS}},H\right)=E\left\{\frac{\left|\right|H_{\mathrm{LS}}-H{\left|\right|}_{F}^{2}}{{\left||H|\right|}_{F}^{2}}\right\},$$

where E{·} expresses the expectation operation; $\left|\right|\cdot {\left|\right|}_{F}^{2}$ means the square of the Frobenius norm.

According to the descending order criterion, the fitness set Fit is divided into $Fit_{\mathrm{fa}}\in R^{Q_{1}\times 1}$, $Fit_{\mathrm{mo}}\in R^{Q_{2}\times 1}$, and $Fit_{\mathrm{su}}\in R^{Q_{3}\times 1}$, where $Q=Q_{1}+Q_{2}+Q_{3}$. The father population corresponding to $Fit_{\mathrm{fa}}$ is defined as $\kappa_{\mathrm{fa}}\in R^{Q_{1}\times M_{\mathrm{acti}}}$, and its gene population is given as $G_{\mathrm{fa}}\in Z^{Q_{1}\times L_{c}\times M_{\mathrm{acti}}}$. The mother-population corresponding to $Fit_{\mathrm{mo}}$ is defined as $\kappa_{\mathrm{mo}}\in R^{Q_{2}\times M_{\mathrm{acti}}}$, and its gene population is given as $G_{\mathrm{mo}}\in Z^{Q_{2}\times L_{c}\times M_{\mathrm{acti}}}$. The sub-population corresponding to $Fit_{\mathrm{su}}$ is defined as $\kappa_{\mathrm{su}}\in R^{Q_{3}\times M_{\mathrm{acti}}}$, and its gene population is given as $G_{\mathrm{su}}\in Z^{Q_{3}\times L_{c}\times M_{\mathrm{acti}}}$.

#### 3.1.3. Crossover

**Figure 2:**
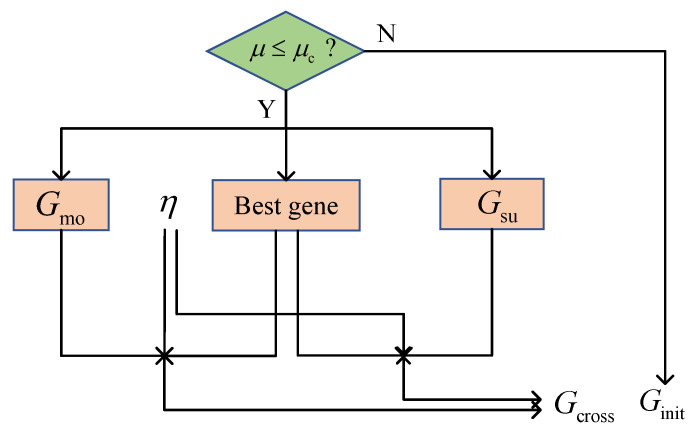
The flowcharts of crossover strategy.

Figure 2 illustrates the proposed crossover strategy. The MMPGA-LS generates a crossover probability μ(0≤μ≤1) and compares it with $\mu_{c}$. The execution of the crossover strategy satisfies a condition, where $\mu \leq \mu_{c}$. Relying on Fit, the best one $\kappa_{\mathrm{best}}\in R^{1\times M_{\mathrm{acti}}}$ is selected as

(23) $$\kappa_{\mathrm{best}}\leftarrow f\left(\max\left\{Fit_{q}\right\}\right),q=1,2,\cdots ,Q.$$

Then new gene populations are generated via $g_{\mathrm{best}}$, $G_{\mathrm{mo}}$, $G_{\mathrm{su}}$, and an index η of the crossover position

(24) $$\begin{array}{cc} \; & g_{2q_{2}-1,m_{acti}}={\left[g_{\mathrm{best}}\left(1,1:\eta \right),g_{\mathrm{mo},q_{2},m_{\mathrm{acti}}}\left(q_{2},\eta +1:L_{c}\right)\right]}^{T},\\ \; & g_{2q_{2},m_{acti}}={\left[g_{\mathrm{mo},q_{2},m_{\mathrm{acti}}}\left(q_{2},1:\eta \right),g_{\mathrm{best}}\left(1,\eta +1:L_{c}\right)\right]}^{T}, \end{array}$$

where $q_{2}=1,2,\cdots ,Q_{2}$,

(25) $$\begin{array}{cc} \; & g_{2q_{3}-1,m_{acti}}={\left[g_{\mathrm{best}}\left(1,1:\eta \right),g_{\mathrm{su},q_{3},m_{\mathrm{acti}}}\left(q_{3},\eta +1:L_{c}\right)\right]}^{T},\\ \; & g_{2q_{3},m_{acti}}={\left[g_{\mathrm{su},q_{3},m_{\mathrm{acti}}}\left(q_{3},1:\eta \right),g_{\mathrm{best}}\left(1,\eta +1:L_{c}\right)\right]}^{T}, \end{array}$$

where $q_{3}=1,2,\cdots ,Q_{3}$. A new gene population $G_{\mathrm{cross}}\in Z^{Q_{4}\times L_{c}\times M_{\mathrm{acti}}}$ is formulated according to Equations (24) and (25), where $Q_{4}=2Q_{2}+2Q_{3}$.

With the substitution of $G_{\mathrm{cross}}$, the corresponding fitness $Fit_{\mathrm{cross}}\in R^{Q_{4}\times 1}$ is obtained. MMPGA-LS abandons the worst one corresponding to the lowest fitness in $G_{\mathrm{cross}}$, reserves $\kappa_{\mathrm{best}}$, and formulates $\kappa_{\mathrm{cross}}\in R^{Q_{4}\times M_{\mathrm{acti}}}$.

#### 3.1.4. Adaptive Mutation

**Figure 3:**
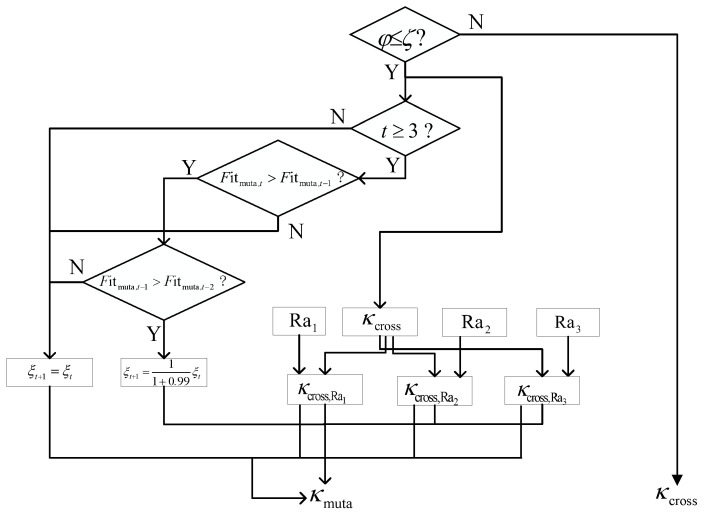
The flowcharts of the adaptive mutation strategy.

Figure 3 represents the flowcharts of the adaptive mutation strategy. The proposed method randomly generates a mutation probability φ(0≤φ≤1) and compares it with ζ. The condition of the adaptive mutation strategy satisfies φ≤ζ. Based on the output of the crossover strategy, three random number sets ${\mathrm{Ra}}_{1},{\mathrm{Ra}}_{2},{\mathrm{Ra}}_{3}\in Z^{Q_{4}\times 1}$ and mutation scale factors ${\left\{\xi_{q_{4}}\right\}}_{q_{4}=1,2,\cdots ,Q_{4}}\in R^{Q_{4}\times 1}$, $\kappa_{\mathrm{muata}}\in R^{Q_{4}\times M_{\mathrm{acti}}}$ are given as

(26) $$\kappa_{\mathrm{muata},q_{4}}=\kappa_{\mathrm{cross},q_{4}}+\xi_{q_{4}}\left(\kappa_{\mathrm{cross},{\mathrm{Ra}}_{1,q_{4}}}-\kappa_{\mathrm{cross},q_{4}}\right)+\xi_{q_{4}}\left(\kappa_{\mathrm{cross},{\mathrm{Ra}}_{2,q_{4}}}-\kappa_{\mathrm{cross},{\mathrm{Ra}}_{3,q_{4}}}\right).$$

Sustain $\kappa_{\mathrm{muata}}$ to construct the corresponding fitness $Fit_{\mathrm{muata}}\in R^{Q_{4}\times 1}$. Compared with the fixed mutation factor, the proposed MMPGA-LS adjusts $\left\{\xi_{q_{4},t}\right\}$.

(27) $$\xi_{q_{4},t+1}=\frac{1}{1+0.99t}\xi_{q_{4},t},Fit_{\mathrm{muata},q_{4},t}>Fit_{\mathrm{muata},q_{4},t-1}\;and\;Fit_{\mathrm{muata},q_{4},t-1}>Fit_{\mathrm{muata},q_{4},t-2},$$

where *t* denotes the number of the current iteration.

#### 3.1.5. Population Reservation

Relying on $Fit_{\mathrm{muata},t}$, the proposed method selects $\kappa_{\mathrm{new},t}\in R^{Q\times M_{\mathrm{acti}}}$ with higher fitness. $G_{\mathrm{new},t}\in Z^{Q\times L_{c}\times M_{\mathrm{acti}}}$ is formulated via the binary transportation about $\kappa_{\mathrm{new},t}$. For an example of $\kappa_{\mathrm{new},t}$, the relationship between $\kappa_{q,t,m_{\mathrm{acti}}}$ and $g_{q,t,m_{\mathrm{acti}}}$ is expressed as

(28) $$g_{q,t,m_{\mathrm{acti}}}=f_{\mathrm{bina}}\left(\left(\frac{\left(\kappa_{q,t,m_{\mathrm{acti}}}-lb\right)2^{L_{c}}}{up-lb}\right)\right\rfloor ),$$

where $f_{\mathrm{bina}}\left(\cdot \right)$ denotes the binary transportation function, and (·)⌋ is an operation of the integral down. The proposed method replaces κ with $\kappa_{\mathrm{new},t}$.

The proposed MMPGA-LS stops the iteration until t>T, where *T* is a number of the total iteration. Collecting the best one in each iteration, we get $\kappa_{\mathrm{iter}}\in R^{T\times M_{\mathrm{acti}}}$ and its corresponding fitness set $Fit_{\mathrm{iter}}\in R^{T\times 1}$. Based on $Fit_{\mathrm{iter}}$, $\kappa^{*}\in R^{1\times M_{\mathrm{acti}}}$ is given as

(29) $$\kappa^{*}\leftarrow f\left(\max{\left\{Fit_{\mathrm{iter}}\right\}}_{t}\right),t=1,\cdots ,T.$$

After using $\kappa^{*}$, the cascaded channel $H_{\mathrm{MMPGA},\mathrm{LS}}$ is obtained via the LS algorithm [35]. To evaluate the performance obtained by the proposed MMPGA-LS, the error function is defined as

(30) $$f\left(H_{\mathrm{MMPGA},\mathrm{LS}},H\right)=E\left\{\frac{\left|\right|H_{\mathrm{MMPGA},\mathrm{LS}}-H{\left|\right|}_{F}^{2}}{{\left||H|\right|}_{F}^{2}}\right\}.$$

#### 3.1.6. ResNet

Based on the output of the proposed MMPGA-LS, the proposed ResNet further reduces the cascaded channel estimation error. The dataset used in the network collects the real part Re{HMMPGA,LS} and the imaginary part Im{HMMPGA,LS} of $H_{\mathrm{MMPGA},\mathrm{LS}}$ to construct $H_{4}\in R^{N_{1}\times N_{2}}$, where $N_{1}N_{2}=2N$.

(31) H4=Re{[HMMPGA,LS]1,1}Im{[HMMPGA,LS]1,1}⋯⋮⋱⋮⋯Re{[HMMPGA,LS]N,1}Im{[HMMPGA,LS]N,1}.

The corresponding operation between the input $H_{4}$ and the output {Re{H′},Im{H′}} is expressed as

**Figure 4:**
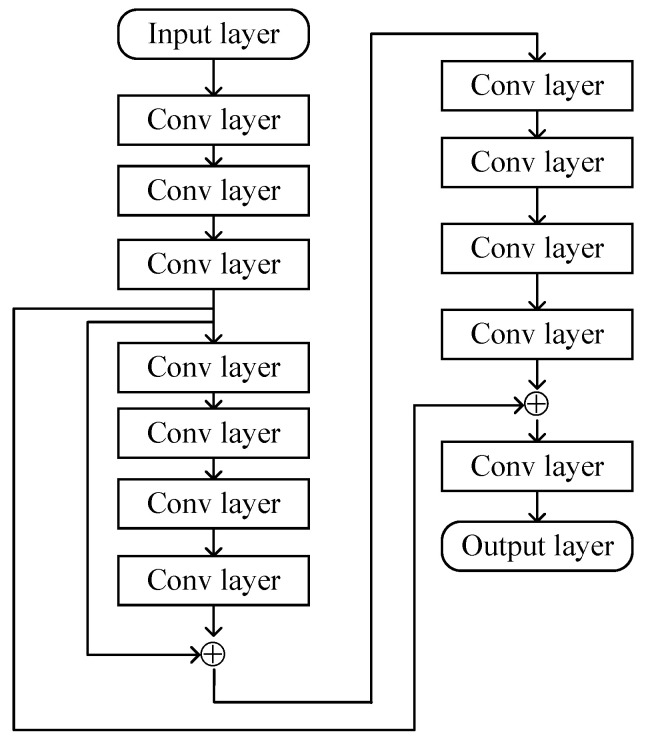
The primary layers of the proposed ResNet.

(32) fL1−1(fL1−1−1⋯(f1−1(H4)))={Re{H′},Im{H′}},

where $L_{1}$ denotes the total layers of ResNet. Figure 4 represents some primary layers of the proposed ResNet. The flowcharts of the cascaded channel estimation based on MMPGA-ResNet are summarized below.

1. Initialize κ;
2. Classify κ based on fitness ranking;
3. Execute crossover operation shown in Figure 2;
4. Execute adaption mutation operation shown in Figure 3;
5. Reserve $\kappa_{\mathrm{new}}$ with the higher fitness;
6. Select the best one $\kappa^{*}$ with the highest fitness;
7. Substitute $\kappa^{*}$ into Equations (1)–(6) and LS algorithm;
8. $H_{4}\leftarrow f\left(H_{\mathrm{MMPGA},\mathrm{LS}}\right)$ based on Equation (31);
9. Substitute $H_{4}$ into the proposed ResNet shown in Figure 4;
10. Train the network parameters in the ResNet;
11. Attain the optimization model of ResNet;
12. $H^{\prime }\leftarrow f\left(H_{4}\right)$.

The first convolution layer extracts the information in $H_{4}$. For each filter used in the convolution layer, the mathematical expression about the input $X\in R^{N_{x}\times N_{y}}\left(0<N_{x}<N_{1},0<N_{y}<N_{2}\right)$ and a 2D convolution kernel with the dimension of $N_{x}\times N_{y}$ is expressed as

(33) $$\left(W^{\left[l_{1}\right]}*X\right)=\sum_{i=1}^{N_{x}}\sum_{j=1}^{N_{y}}W_{i,j}^{\left[l_{1}\right]}X_{i,j},$$

where $W^{\left[l_{1}\right]}$ means the weight of a kernel in the $l_{1}$th layer. The corresponding bias *b* is added to $\left(W^{\left[l_{1}\right]}*X\right)$, where *b* is an element of $b\in R^{F_{c}\times 1}$, and $F_{c}$ is the number of filters. The convolution layer selects the filter with a size of 3×3. Following each convolution layer, the activation function selects the LeakyReLU function, which is expressed as

(34) $$y=\left\{\begin{array}{cc} \; & x,x\geq 0\\ \; & \iota x,x<0 \end{array}\right..$$

Furthermore, the network utilizes the batch-normalization operation to avoid the over-fit [36]. In ${\left\{f^{-1}\right\}}_{i=2,6,11,15}$, we select 64 filters. The convolution layers in ${\left\{f^{-1}\right\}}_{i=3,7,12}$ deploy 128 filters to further process the data from the current input. The operations in ${\left\{f^{-1}\right\}}_{i=4,8,13}$ select 256 filters. The convolution layers in ${\left\{f^{-1}\right\}}_{i=5,10}$ use 32 filters. Following ${\left\{f^{-1}\right\}}_{4}$, the ResNet processes the corresponding output in parallel. In ${\left\{f^{-1}\right\}}_{9}$, the proposed ResNet implements the cross-layers operation by adding the outputs of ${\left\{f^{-1}\right\}}_{4}$ and ${\left\{f^{-1}\right\}}_{8}$. The cross-layers operation is also done in ${\left\{f^{-1}\right\}}_{14}$ by adding the outputs of ${\left\{f^{-1}\right\}}_{4}$ and ${\left\{f^{-1}\right\}}_{13}$. The classical InceptionNets [37] also uses the cross-layers operation to improve learning ability.

The flattening operation used in ${\left\{f^{-1}\right\}}_{16}$ transforms the 2D matrix obtained by the last convolution layer into a column scalar. Finally, the hidden layer, with multiple neurons in the output layer, processes the column scalar. The corresponding mathematical operation between the current input x and output z is given as

(35) $$z=W_{\mathrm{hidd}}x+b_{\mathrm{hidd}}.$$

In the output layer, the proposed ResNet predicts the real and imaginary parts of the cascaded channel. Subsequently, (Re{H′},Im{H′}) reformulates the predicted channel $H^{\prime }$, which is expressed as H′=Re{H′}+jIm{H′}.

To evaluate the estimation performance achieved by the ResNet, the NMSE function is used as the error function.

(36) $$f_{\mathrm{NMSE}}\left(H^{\prime },H\right)=E\left\{\frac{\left|\right|H^{\prime }-H{\left|\right|}_{F}^{2}}{{\left||H|\right|}_{F}^{2}}\right\}.,$$

Based on the gradient descent algorithm, learning rate $r_{1}$, $f_{\mathrm{NMSE}}\left(H^{\prime },H\right)$, and momentum factors, the ResNet updates the prediction. Table 1 represents the configuration of some primary layers in the ResNet.

### 3.2. MTRnet-Based Reflected Channel Estimation

Based on the output of the ResNet, the proposed MTRnet achieves the reflected channel estimation in the second step. The mapping between input (Re{H′},Im{H′}) and output $\left(\theta^{\prime },\omega^{\prime },\phi^{\prime },\alpha_{\mathrm{BS}}^{\prime },\alpha_{\mathrm{RIS}}^{\prime }\right)$ is expressed as

**Figure 5:**
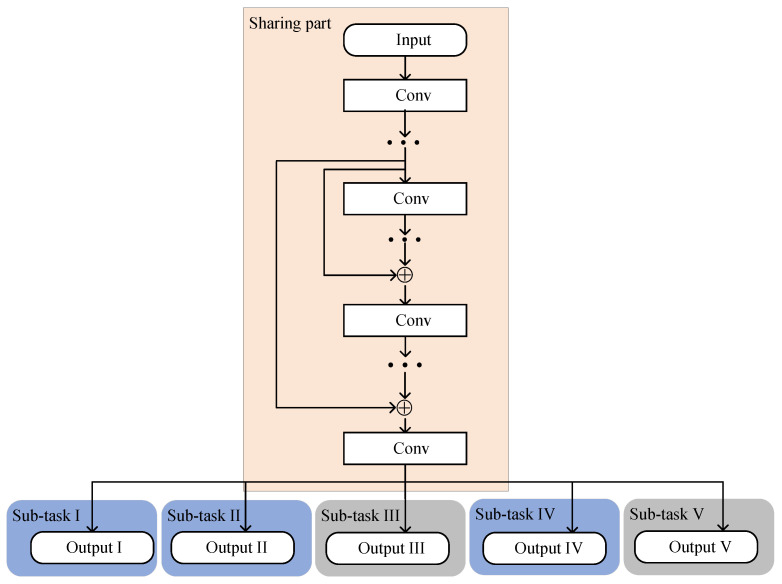
Network structure of the proposed MTRnet.

(37) fL2−1(fL2−1−1⋯(f1−1(Re{H′},Im{H′}))={θ′,ω′,ϕ′,αBS′,αRIS′},

where $L_{2}$ means the total layers of the MTRnet. Based on Equations (2)–(6), there exists a relationship between $\left(\theta^{\prime },\omega^{\prime },\phi^{\prime },\alpha_{\mathrm{BS}}^{\prime },\alpha_{\mathrm{RIS}}^{\prime }\right)$ and $\left(H_{1}^{\prime },H_{2}^{\prime }\right)$. Figure 5 represents the network structure of the proposed MTRnet.

The MTRnet mainly contains a sharing part and multiple sub-tasks. The sharing part implements some convolution layers. Except for the output layer, the convolution layers in the sharing part are the same as the ResNet. Considering the 2D convolution kernel, (Re{H′},Im{H′}) is firstly reshaped into $H_{5}\in R^{N_{1}\times N_{2}}$.

(38) H5=Re{[H′]1,1}Im{[H′]1,1}⋯⋮⋱⋮⋯Re{[H′]N,1}Im{[H′]N,1}.

The MTRnet selects the output of the sharing part as the input for all sub-tasks. Following the last convolution-activation-batch-normalization layers in the sharing part, five sub-tasks further process the current input simultaneously. Remarkably, each sub-task has its own exclusive training parameters. In sub-task I and sub-task II, the corresponding output layers both select *P* neurons to generate $\left\{\theta^{\prime },\omega^{\prime }\right\}$. Meanwhile, the third sub-task with 2P neurons learns the mapping between the current input and (Re{α1′},Im{α1′}). α1′=Re{α1′}+jIm{α1′}. The fourth sub-task with *P* neurons achieves the prediction of $\phi^{\prime }$. The output layer in the fifth sub-task utilizes 2P neurons to generate (Re{α2′},Im{α2′}) and formulate $\alpha_{2}^{\prime }$. The Tanh activation function is used as the activation function in the second network, which is expressed as $f_{\mathrm{Tanh}}\left(x\right)=\frac{e^{x}-e^{-x}}{e^{x}+e^{-x}}$. The proposed multi-task regression network selects the mean square error function and Adam optimizer to update the network parameters. Table 2 shows the configuration of network parameters in the five sub-tasks.

Finally, the proposed method achieves the reflected channel estimation through the mapping between outputs obtained by the multi-task regression network and $\left(H_{1}^{\prime },H_{2}^{\prime }\right)$. It is clear that $H_{1}^{\prime }\leftarrow f\left(\theta^{\prime },\omega^{\prime },\alpha_{1}^{\prime }\right)$ and $H_{2}^{\prime }\leftarrow f\left(\phi^{\prime },\alpha_{2}^{\prime }\right)$. To evaluate the estimation performance in the reflected channels, we also select the NMSE function.

(39) $$f_{\mathrm{NMSE}}\left(H_{1}^{\prime },H_{1}\right)=E\left\{\frac{\left|\right|H_{1}^{\prime }-H_{1}{\left|\right|}_{F}^{2}}{\left|\right|H_{1}{\left|\right|}_{F}^{2}}\right\},$$

(40) $$f_{\mathrm{NMSE}}\left(H_{2}^{\prime },H_{2}\right)=E\left\{\frac{\left|\right|H_{2}^{\prime }-H_{2}{\left|\right|}_{F}^{2}}{\left|\right|H_{2}{\left|\right|}_{F}^{2}}\right\}.$$

### 3.3. Implementation of the Novel Two-Step Method

The proposed method is decomposed into two steps, including the MMPGA-LS-ResNet-based cascaded channel estimation and the MTRnet-based reflected channel estimation. In the cascaded channel estimation, the proposed MMPGA-LS first generates the population $\kappa ,G_{\mathrm{init}}$. Then, the proposed MMPGA attains $\kappa^{*}$ via the population classification, crossover operation, adaptive mutation operation, and population reservation. Based on $\kappa^{*}$ and the LS estimator, $H_{4}$ is formulated from $H_{\mathrm{MMPGA},\mathrm{LS}}$. Furthermore, the proposed method achieves the cascaded channel estimation via learning the relationship between the cascaded channel obtained by MMPGA-LS and the channel of UE-RIS-BS. $H_{4}$ is used as the input of the proposed ResNet. The corresponding output in the ResNet is expressed as $H^{\prime }$. As a strong de-noise ability, the ResNet is capable of further reducing the cascaded channel estimation error. The output of the ResNet is reshaped into $H_{5}$ and used as the input to the proposed MTRnet. The MTRnet using a multi-task regression model and ResNet estimates the channels of RIS-BS and UE-RIS simultaneously. As a result, the channel parameters $\left(\alpha_{1}^{\prime },\theta^{\prime },\omega^{\prime },\alpha_{2}^{\prime },\phi^{\prime }\right)$ are used as the output of the MTRnet. Finally, the proposed method reconstructs the reflected channels based on the output of MTRnet.

**Figure 6:**
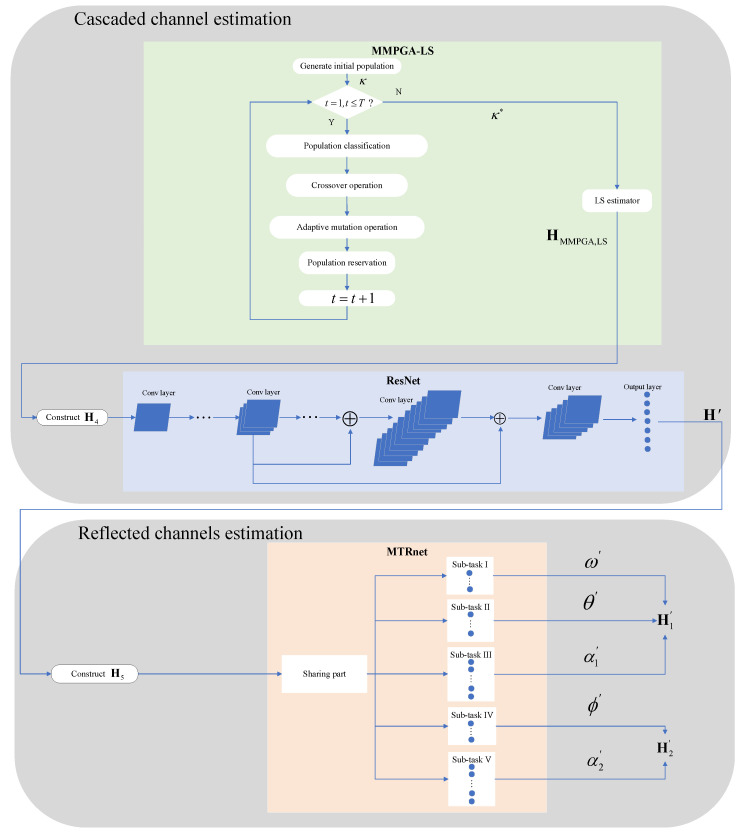
The flowcharts of the novel two-step method.

The flowcharts of the proposed two-step method are shown in Figure 6, which can be summarized as follows:

1. Generate population $\kappa ,G_{\mathrm{init}}$;
2. Attain $\kappa^{*}$ via the proposed MMPGA;
3. Obtain $H_{\mathrm{MMPGA},\mathrm{LS}}$ by LS estimator;
4. $H_{4}\leftarrow f\left(H_{\mathrm{MMPGA},\mathrm{LS}}\right)$;
5. Substitute $H_{4}$ into the proposed ResNet;
6. Generate $H^{\prime }$ via the optimization model of ResNet;
7. $H_{5}\leftarrow f\left(H^{\prime }\right)$;
8. Substitute $H_{5}$ into the proposed MTRnet;
9. Achieve $\left(\alpha_{1}^{\prime },\theta^{\prime },\omega^{\prime },\alpha_{2}^{\prime },\phi^{\prime }\right)$ via the optimization model of MTRnet;
10. $H_{1}^{\prime }\leftarrow f\left(\theta^{\prime },\omega^{\prime },\alpha_{1}^{\prime }\right)$, $H_{2}^{\prime }\leftarrow f\left(\phi^{\prime },\alpha_{2}^{\prime }\right)$.

In Figure 6, the light green part represents the MMPGA-LS. The portion with light blue stand for the ResNet. The beige colored part of Figure 6 denotes the MTRnet.

With much discussion of the cascaded channel estimation, the proposed MMPGA-LS-ResNet can be summarized as follows:

1. Initialize population;
2. Classify $\kappa ,G_{\mathrm{init}}$ via the descending order criterion of fitness;
3. Execute the crossover operation based on Equations (24) and (25);
4. Adopt the adaptive mutation strategy based on Equations (26) and (27);
5. Select $\kappa_{\mathrm{new},t}$ with higher fitness;
6. Replace κ with $\kappa_{\mathrm{new},t}$;
7. Replace $G_{\mathrm{init}}$ with $G_{\mathrm{new}}$;
8. Estimate $H_{\mathrm{MMPGA},\mathrm{LS}}$;
9. Construct $H_{4}$;
10. Predict $H^{\prime }$ via the optimization model of ResNet.

As a result, $H^{\prime }$ is exported and used as the input to the MTRnet. Then, after much discussion of reflected channel estimation, the MTRnet-based method can be summarized as follows:

1. Construct $H_{5}$;
2. Predict $H_{1}^{\prime }$ and $H_{2}^{\prime }$ by the optimization model of MTRnet;
3. Evaluate the channel estimation performance based on Equations (39) and (40).

## 4. Simulation Results and Discussion

In this section, a series of results validate the superiority of the proposed method. The SNR regimes contain {0,5,10,15,20} dB. In each SNR, the network uses a dataset with a length of 110,000. The length of the training dataset is 90000, and the validation dataset contains the dataset with a length of 10,000. The remaining data belong to the test dataset. Throughout the simulations, the RIS implements the formulation of the uniform linear array with M=32 elements and $M_{\mathrm{acti}}=4$. The BS uses the uniform linear array with N=16 elements, $L_{s}\in \left\{16,32,64\right\}$, P=4, and the half-wavelength spacing. Besides, we select $Q=100,\;L_{c}=20,\;Q_{1}=20,\;Q_{2}=20,\;Q_{3}=60,\;\mu_{1}=0.8,\;\xi =0.25,\;T=200$. Keras 2.2 is used to implement the proposed neural networks. The networks are running on Python 3.5, cuda 10.0, cuDNN 7.6, and GPU 8G. MMPGA-LS is compared with GA [29] and PSO [30] in terms of cascaded channel estimation NMSE. For the reflected channel estimation performance, CNN [15] and CRNN [17] are compared with the proposed method in terms of the NMSE.

### 4.1. Comparisons with Reported Methods

Table 3 summarizes the computation complexity of different methods. The computation in the proposed MMPGA is mainly concentrated on initialization, crossover, and mutation operations. The computation complexity of the initialization is about the population $Q^{2}$ and the complexity $O(NL_{s}^{4})$ of the LS algorithm, which is expressed as $O(M_{\mathrm{acti}}Q^{2}NL_{s}^{4})$. The computational complexity of the crossover operation is proportional to $Q_{4}$ and the LS algorithm, which is shown as $O(M_{\mathrm{acti}}Q_{4}NL_{s}^{4})$. The computational complexity of the mutation is proportional to $Q_{4}$$L_{c}$ and $O(NL_{s}^{4})$, and its computational complexity is denoted as $O(M_{\mathrm{acti}}Q_{4}L_{c}NL_{s}^{4})$. As a result, the complexity of the proposed MMPGA is written as $O(TM_{\mathrm{acti}}NL_{s}^{4}\left(Q^{2}+Q_{4}+Q_{4}L_{c}\right))$. The computational complexity of PSO [30] is $O(2M_{\mathrm{acti}}TQNL_{s}^{4})$. The computational complexity of GA [29] stands for $O(TM_{\mathrm{acti}}NL_{s}^{4}\left(Q^{2}+2Q\right))$. The training parameters are widely used for evaluating the computational complexity of the neuron network. In the convolution layer, the training parameters are expressed as $\left(N_{x}N_{y}C_{\mathrm{in}}+1\right)C_{\mathrm{out}}$, where $C_{\mathrm{in}}$ means the filter numbers in the current layer and $C_{\mathrm{out}}$ are the filter numbers in the next layer [38]. The training parameters in the hidden layer are denoted as $\left(D_{\mathrm{in}}+1\right)D_{\mathrm{out}}$, where $D_{\mathrm{in}}$ stands for the neurons number in the current layer and $D_{\mathrm{out}}$ is denoted as the neurons number in the next layer [38]. According to Table 1 and Figure 5, the computational complexity of the proposed neuron networks is expressed as $\sum_{l_{c}=1}^{L_{\mathrm{conv}}-1}\left(N_{x}N_{y}C_{\mathrm{in}}+1\right)C_{\mathrm{out}}+\sum_{l_{d}=1}^{L_{\mathrm{hidd}}-1}\left(D_{\mathrm{in}}+1\right)D_{\mathrm{out}}$. The computational complexity of CRNN [17] stands for $\sum_{l_{c}=1}^{L_{\mathrm{CRNN},\mathrm{conv}}-1}\left(P+3\right)\left(N_{\mathrm{CRNN},x}N_{\mathrm{CRNN},y}C_{\mathrm{CRNN},\mathrm{in}}+1\right)C_{\mathrm{CRNN},\mathrm{out}}+\sum_{l_{d}=1}^{L_{\mathrm{CRNN},\mathrm{hidd}}-1}\left(D_{\mathrm{CRNN},\mathrm{in}}+1\right)D_{\mathrm{CRNN},\mathrm{out}}$, where $\left(N_{\mathrm{CRNN},x},N_{\mathrm{CRNN},y}\right)$ means the 2D dimension of filters, $L_{\mathrm{CRNN},\mathrm{conv}}$ is the number of the convolution layers, and $L_{\mathrm{CRNN},\mathrm{hidd}}$ stands for the number of hidden layers. The computational complexity of CNN [15] is $\sum_{l_{c}=1}^{L_{\mathrm{CNN},\mathrm{conv}}-1}\left(P+3\right)\left(N_{\mathrm{CNN},x}N_{\mathrm{CNN},y}C_{\mathrm{CNN},\mathrm{in}}+1\right)C_{\mathrm{CNN},\mathrm{out}}+\sum_{l_{d}=1}^{L_{\mathrm{CNN},\mathrm{hidd}}-1}\left(D_{\mathrm{CNN},\mathrm{in}}+1\right)$.

**Figure 7:**
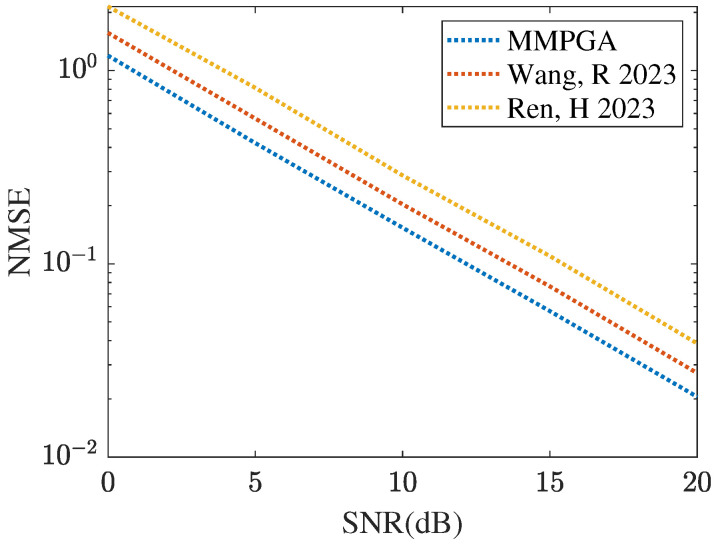
Cascaded channel estimation performance obtained by different evolution algorithms.

Figure 7 represents the cascaded channel estimation performance obtained by different heuristic algorithms, including GA [29], PSO [30], and the proposed MMPGA-LS. As shown in Figure 7, the proposed MMPGA-LS achieves a lower channel estimation compared with that achieved by GA [29] and PSO [30]. In SNR 20 dB, the MMPGA-LS obtains the NMSE of 0.0205, which is lower than 0.0272 achieved by PSO [30] and 0.0387 achieved by GA [29]. Compared with GA [29], the proposed MMPGA-LS abandons the random operation in the crossover operation and adaptively changes the factors in the mutation operation. As a result, the proposed MMPGA-LS is capable of further reducing the channel estimation error. It is concluded from Figure 7 that the proposed MMPGA-LS outperforms GA [29] and PSO [30] in terms of a lower NMSE.

**Figure 8:**
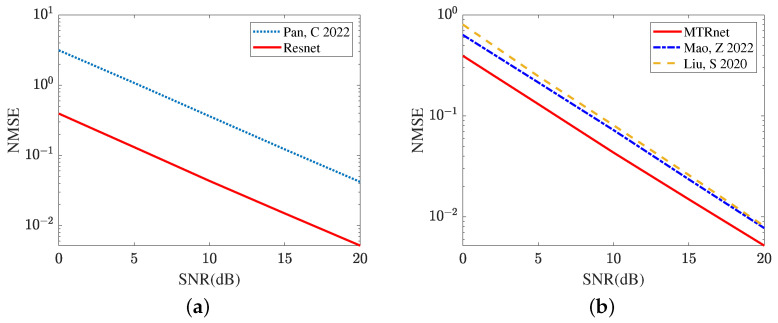
Cascaded channel estimation performance comparison by different models. (**a**) Cascaded channel estimation performance comparison between the deep learning and model-driven. (**b**) Cascaded channel estimation performance comparison among different deep learning models.

In the next sub-simulation, we compare the cascaded channel estimation performance achieved by the traditional algorithm and some existing deep-learning-based methods. Figure 8a compares the channel estimation NMSE in the LS algorithm [35] and the proposed ResNet. The ResNet, with its strong de-noise ability, effectively suppresses the inference from the noise. Relying on the curves plotted in Figure 8a, the proposed ResNet obtains a lower NMSE across a range of SNR regimes. In Figure 8b, we compare the cascaded channel estimation NMSE obtained by CRNN [17], CNN [15], and the proposed ResNet. It is clear that the deep-learning-based methods significantly reduce the NMSE compared with the LS algorithm [35]. In SNR 20 dB, the ResNet achieves NMSE 0.0052, which is reduced by 32.47% relative to CRNN [17] and 36.25% relative to CNN [15]. The proposed ResNet with the cross-layers operation explores the relationship between different layers and has a stronger learning ability compared with CRNN [17] and CNN [15]. Therefore, the proposed ResNet can further reduce the cascaded channel estimation NMSE. It is concluded from Figure 8 that the proposed ResNet is superior to the LS algorithm [35], CRNN [17], and CNN [15].

**Figure 9:**
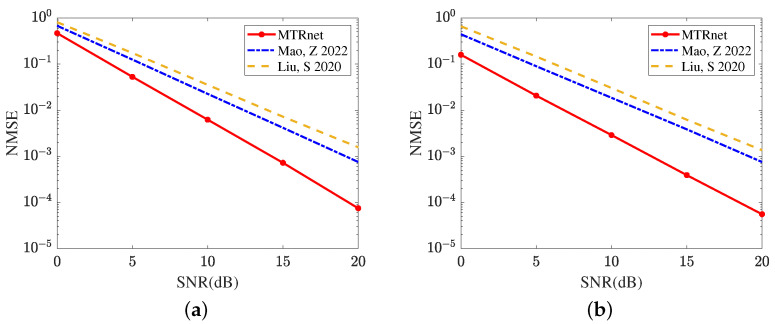
Reflected channel estimation performance comparison in different deep learning models (**a**) Channel estimation NMSE of RIS-BS. (**b**) Channel estimation NMSE of UE-RIS.

Figure 9 compares the reflected channel estimation performance obtained by different deep learning models, including CRNN [17], CNN [15], and the proposed MTRnet. Figure 9a evaluates the channel estimation NMSE of RIS-BS. As the single output layer, CRNN [17] and CNN [15] both use multiple optimization models to achieve this channel estimation. Remarkably, the proposed MTRnet with multiple output layers only requires one model. In SNR 20 dB, the proposed MTRnet obtains an NMSE of $7.4688\times 10^{-5}$, which is lower than $7.5237\times 10^{-4}$ achieved by CRNN [17] and $1.5689\times 10^{-3}$ achieved by CNN [15]. Figure 9b exhibits the channel estimation NMSE of UE-RIS. The proposed MTRnet also obtains a lower NMSE compared with CRNN [17] and CNN [15]. In SNR 0 dB, the proposed MTRnet obtains an NMSE of 0.1587, which is lower than 0.4415 achieved by CRNN [17] and 0.6581 achieved by CNN [15]. It is observed from Figure 9 that the proposed MTRnet can achieve the lower reflected channel estimation NMSE simultaneously.

### 4.2. Numerical Results of The Proposed Two-Step Mehod

**Figure 10:**
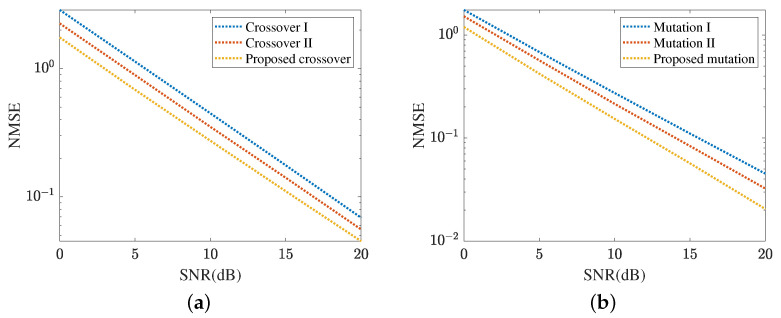
Cascaded channel estimation performance of the proposed MMPGA-LS. (**a**) Performance comparison in different crossover strategies. (**b**) Performance comparison in different mutation strategies.

Figure 10 demonstrates the cascaded channel estimation NMSE obtained by the proposed MMPGA-LS. In Figure 10a, we investigate the performance comparison with three crossover strategies. In crossover I, one randomly selected child of the father population, mother population, and sub-population execute the crossover operation. Differently, children in the father population and mother population are paired in descending order of fitness and perform crossover operations in turn. Besides, one randomly selected child of the father population and sub-population execute the crossover operation in the crossover II. As shown in Figure 10a, the proposed crossover strategy achieves a lower NMSE compared with crossover I and crossover II. The NMSE obtained by the crossover II is minor. The crossover I achieves the highest NMSE. The proposed crossover strategy takes advantage of the best one with the highest fitness and is conducive to reducing the cascaded channel estimation error. In SNR 20 dB, the proposed crossover strategy obtains an NMSE of 0.0456, which is lower than 0.05579 achieved by crossover II and 0.06886 achieved by crossover I. Figure 10b compares the cascaded channel estimation NMSE in different mutation strategies. In mutation I, this mutation is based on the binary children, and a random position corresponding to each gene is changed to 0/1. Mutation II uses the decimal children and a fixed scale factor. The cascaded channel estimation performance in mutation I is sensitive to the length and requires a sufficiently long sequence to achieve a low NMSE. The proposed mutation strategy can adjust the scale factor set according to different fitness levels. As a result, the proposed mutation strategy reduces cascaded channel estimation NMSE compared with mutation I and mutation II. In SNR 20 dB, the proposed mutation strategy obtains an NMSE of 0.0205, which is lower than 0.0324 achieved by mutation II and 0.0453 achieved by mutation I.

**Figure 11:**
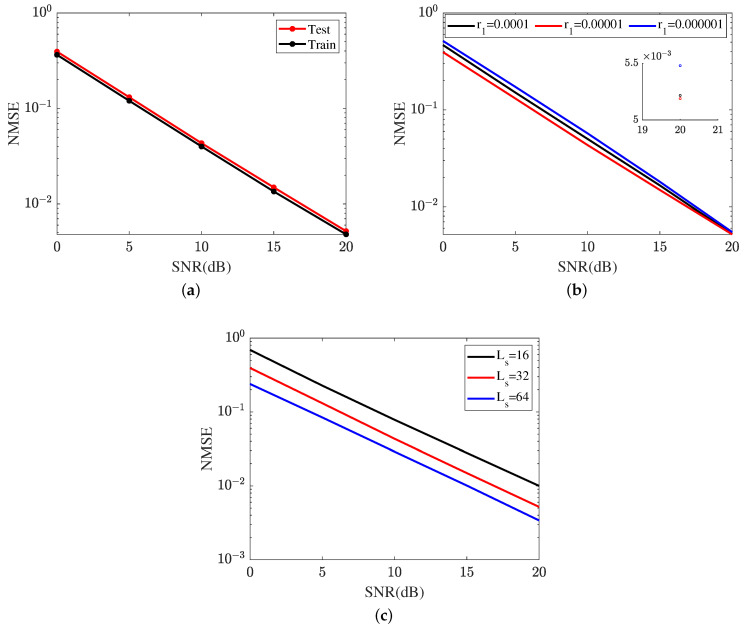
Cascaded channel estimation performance of the proposed ResNet. (**a**) Cascaded channel estimation performance in different datasets. (**b**) Cascaded channel estimation performance at different learning rates. (**c**) Cascaded channel estimation performance in different lengths of signal sequence.

Figure 11 represents the cascaded channel estimation performance achieved by the proposed ResNet. In Figure 11a, we compare the cascaded channel estimation NMSE in the training dataset and test dataset. As shown in Figure 11a, the proposed ResNet achieves a lower NMSE in the training dataset compared with that in the test dataset. Figure 11b investigates the impact of different learning rates on the cascaded channel estimation performance, including $r_{1}\in \left\{0.0001,0.00001,0.000001\right\}$. The learning rate is one of the key parameters in neural network optimization and has an important influence on the learning ability of the ResNet. The ResNet achieves the lowest NMSE in $r_{1}=0.00001$. The NMSE in $r_{1}=0.000001$ is minor. Based on the curves plotted in Figure 11b, the proposed ResNet selects $r_{1}=0.00001$ in terms of a lower cascaded channel estimation NMSE. In Figure 11c, we investigate the impact of different lengths of signal sequence on the cascaded channel estimation performance, including $L_{s}\in \left\{16,32,64\right\}$. In SNR 20 dB, the proposed ResNet obtains an NMSE of 0.0052 in $L_{s}=32$, which is higher than 0.0034 in $L_{s}=64$ and lower than 0.01 in $L_{s}=16$. Relying on the result shown in Figure 11c, the cascaded channel estimation NMSE reduces as the length of the signal sequence increases.

**Figure 12:**
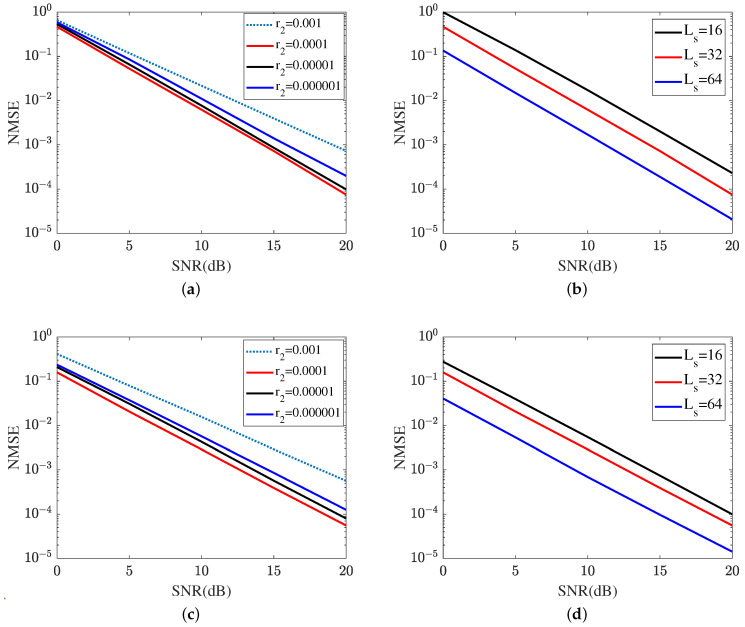
Reflected channel estimation is achieved by the proposed MTRnet. (**a**) Reflected channel estimation of RIS-BS at different learning rates. (**b**) Reflected channel estimation of RIS-BS at different lengths of signal sequence. (**c**) Reflected channel estimation of UE-RIS at different learning rates. (**d**) Reflected channel estimation of UE-RIS at different lengths of signal sequence.

Figure 12 shows the reflected channel estimation performance achieved by the proposed MTRnet. Figure 12a,b represent the reflected channel estimation NMSE of RIS-BS. As shown in Figure 12a, the MTRnet with $r_{2}=0.0001$ attains the lowest NSME within the same iteration. In SNR 20 dB, the proposed ResNet with $r_{2}=0.0001$ obtains an NMSE of $7.4690\times 10^{-5}$, which is lower than $9.8017\times 10^{-5}$ in $r_{2}=0.00001$ and $1.9822\times 10^{-4}$ in $r_{2}=0.00001$, and $7.2888\times 10^{-4}$ in $r_{2}=0.001$. Therefore, $r_{2}=0.0001$ is applied for the next sub-simulations. In Figure 12b, we investigate the impact of different lengths of signal sequence on the cascaded channel estimation performance of RIS-BS. The reflected channel estimation NMSE versus the growth of signal sequence reduces. Figure 12c,d represent the reflected channel estimation NMSE of UE-RIS. Figure 12c represents the impact of different learning rates on the reflected channel estimation performance of UE-RIS, including $r_{2}\in \left\{0.001,0.0001,0.00001,0.000001\right\}$. Based on the result plotted in Figure 12c, the proposed MRRnet with $r_{2}=0.0001$ also attains the lowest NMSE in the reflected channel of UR-RIS. As a result, the proposed MTRnet selects the learning rate $r_{2}=0.0001$. Figure 12d illustrates the reflected channel estimation performance in $L_{s}\in \left\{16,32,64\right\}$. As shown in Figure 12d, the proposed MTRnet achieves the highest NMSE in $L_{s}=16$. In SNR 20 dB, the proposed ResNet obtains an NMSE of $1.4192\times 10^{-5}$ in $L_{s}=64$, which is lower than $5.5447\times 10^{-5}$ in $L_{s}=32$ and $9.9193\times 10^{-5}$ in $L_{s}=16$. It is concluded from Figure 11c and Figure 12b,d that the channel estimation NMSE reduces as the length of the signal sequence grows.

### 4.3. Discussion of the Proposed Method

In the cascaded channel estimation problem, the proposed MMPGA-LS is capable of generating more children compared to GA [29], which does contribute to reducing the cascaded channel estimation NMSE. Besides, MMPGA-LS does not select one randomly and makes good use of the best gene during the crossover operation. The corresponding result shown in Figure 10a has validated the effectiveness of the proposed crossover strategy. Furthermore, the MMPGA-LS refers to the fitness and adjusts the scale factor during the adaptive mutation operation compared with the fixed factor in common evolution algorithms. Therefore, the proposed MMPGA-LS is capable of achieving a lower NMSE. The proposed ResNet with the cross-layers operation and key parameter optimization has a stronger non-linear processing ability compared with CRNN [17] and CNN [15]. As a result, the proposed ResNet with the designed network architecture can attain a lower cascaded channel estimation NMSE compared with CRNN [17] and CNN [15].

For the reflected channels estimation, the proposed MTRnet, integrating the multi-task regression model and ResNet, is introduced. The MTRnet with multiple output layers has a smaller number of network trainings. This is because the MTRnet abandons repetitive network training compared with the single regression model. However, CRNN [17] and CNN [15] with the single output layer require multiple optimization models to estimate the reflected channels. Additionally, the proposed MTRnet obtains a lower reflected channel estimation NMSE compared with CRNN [17] and CNN [15].

**Figure 13:**
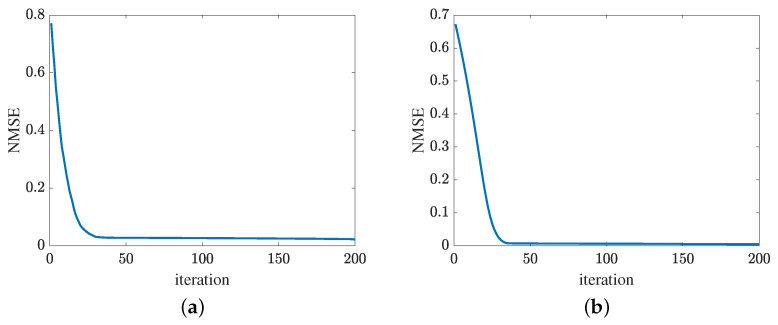
Cascaded channel estimation performance. (**a**) Convergence of the proposed MMPGA. (**b**) Convergence of the proposed ResNet.

**Figure 14:**
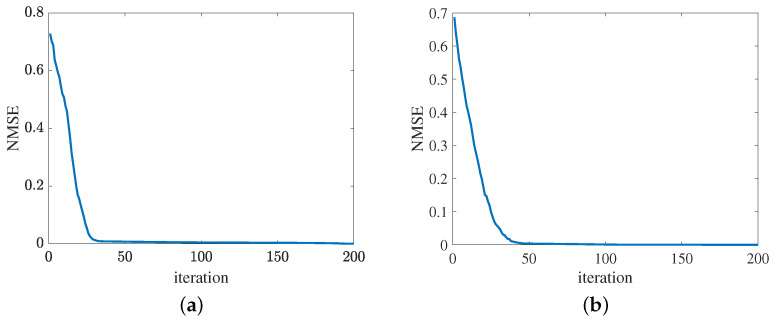
Convergence of reflected channel estimation performance. (**a**) IRS-BS channel estimation. (**b**) UE-RIS channel estimation.

Figure 13 presents the convergence of the proposed method in terms of the cascaded channel estimation performance. As shown in Figure 13a, the NMSE versus the growth of iterations decreases. Besides, the error obtained by the proposed MMPGA slightly decreases in later iterations. In Figure 13b, the proposed ResNet also represents the same tendency as the MMPGA. It is concluded from Figure 13, the proposed method has good convergence on the cascaded channel estimation. The convergence of reflected channel estimation performance is shown in Figure 14. As observed from Figure 14a, the channel estimation error decreases as the neuron network with the gradient descent optimization trains. At NMSE $7.47\times 10^{-5}$, the channel estimation performance of RIS-BS obtained by the MTRnet decreases slowly. Figure 14b exhibits the convergence of UE-RIS channel estimation performance. The tendency of reflected channel estimation performance degradation can also be seen in Figure 14b. It is clear from Figure 14 that the proposed method has good convergence in terms of the reflected estimation NMSE.

**Figure 15:**
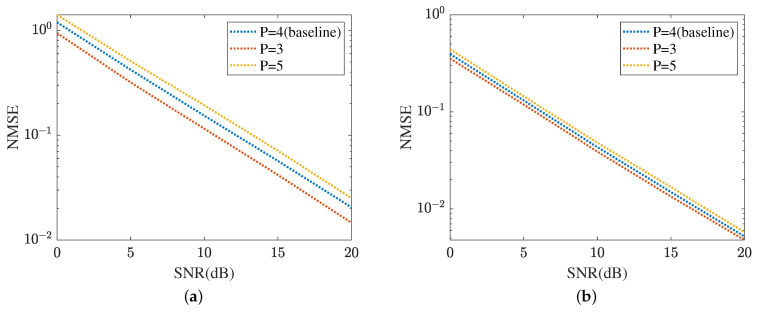
Cascaded channel estimation performance. (**a**) Robustness of the proposed MMPGA. (**b**) Robustness of the proposed ResNet.

**Figure 16:**
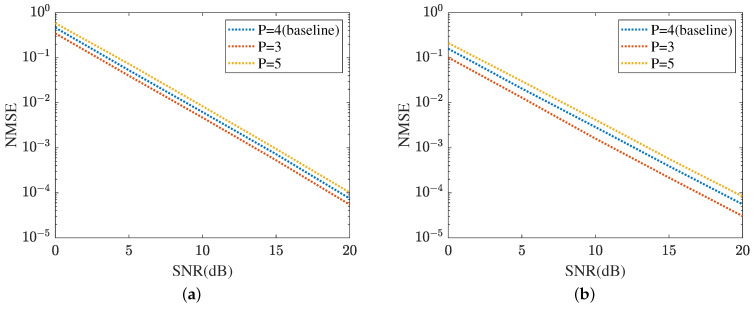
Robustness of reflected channels estimation performance. (**a**) IRS-BS channel estimation. (**b**) UE-RIS channel estimation.

The robustness of the proposed method is shown in Figure 15 and Figure 16, respectively. Figure 15 displays the robustness of the MMPGA and ResNet, where P∈{3,4,5}. The proposed method selects P=4 as the baseline. As shown in Figure 15a, the NMSE in P=3 obtained by the MMPGA is lower than that in P=4. This is because the cascaded channel in P=3 is associated with fewer channel parameters. Therefore, the channel parameters in P=3 can be simply seen as a subset of those in P=4. As a result, the proposed method is capable of obtaining a lower NMSE in P=3 compared with that in P=4. In SNR=20 dB, the MMPGA achieves an NMSE of 0.0146, which is lower than 0.0205 in P=4. Figure 15b represents the robustness of the proposed ResNet in terms of the cascaded channel estimation performance. The model is trained in P=4 and tested in P∈{3,5}. The NMSE in P=5 is slightly higher than that in P=4. The proposed ResNet has less robustness in P=5. Due to the increased channel parameters, the ResNet can adjust the network architecture to obtain a lower NMSE. The robustness of the MTRnet is also shown in Figure 16. Figure 16a evaluates the robustness of the proposed MTRnet in terms of the RIS-BS channel. The proposed method has good robustness in P=3. Besides, Figure 16b shows the robustness of the proposed MTRnet in terms of the UE-RIS channel. The proposed method has less robustness in P=5.

**Figure 17:**
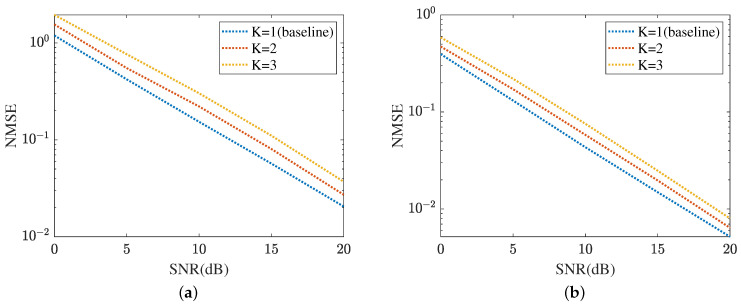
Cascaded channel estimation performance under varying levels of interference. (**a**) MMPGA. (**b**) ResNet.

**Figure 18:**
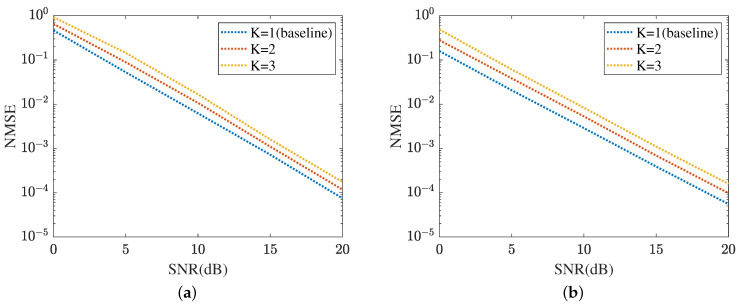
Reflected channel estimation performance under varying levels of interference. (**a**) IRS-BS channel estimation. (**b**) UE-RIS channel estimation.

Figure 17 displays the cascaded channel estimation under varying levels of interference, where K∈{1,2,3}, *K* means the number of UEs. The proposed method selects K=1 as the baseline. Based on the curves plotted in Figure 17a, the cascaded channel estimation NMSE versus the growth of interference level decreased. This is because the cascaded channel performance deteriorates with the interference among the multipath signals, including multi-UEs. Figure 17b evaluates the robustness of the proposed ResNet in terms of varying levels of interference. In SNR=20 dB, the proposed ResNet obtains the lowest NMSE in K=1 and the highest NMSE in K=3. The NMSE in K=2 is minor. Figure 18 summarizes the reflected channel estimation performance under varying levels of interference. The tendency shown in Figure 17 is also represented in Figure 18. Figure 18a focuses on the reflected channel of RIS-BS. Differently, Figure 18b represents the UE-RIS channel estimation performance in terms of varying levels of interference. The proposed MTRnet achieves an NMSE of $9.79\times 10^{-5}$ in K=2, which is slightly higher than $5.54\times 10^{-5}$ in K=1 and lower than 0.00016 in K=3. According to Figure 17 and Figure 18, the proposed method can achieve a low NMSE under varying levels of interference.

**Figure 19:**
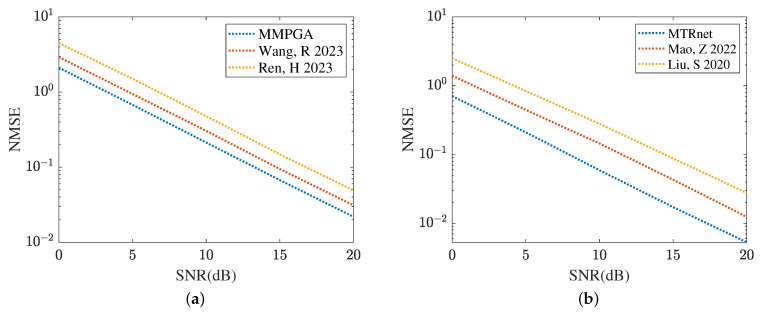
Cascaded channel estimation performance in UPA. (**a**) Comparison of different heuristic algorithms. (**b**) Comparison of different learning models.

**Figure 20:**
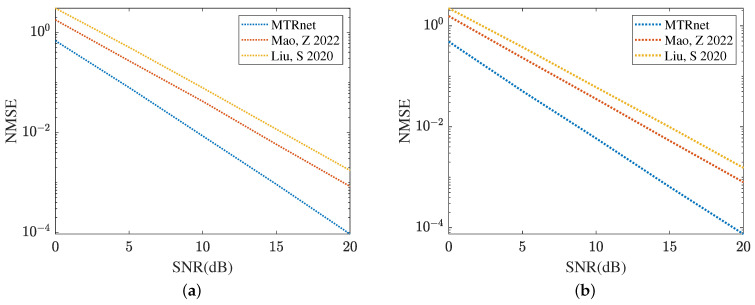
Reflected channels estimation performance comparison in UPA. (**a**) IRS-BS channel estimation. (**b**) UE-RIS channel estimation.

Figure 19 and Figure 20 compare the channel estimation performance based on different models in the RIS with the formulation of UPA, where $M_{x}=M_{y}=8$. As shown in Figure 19a, the proposed MMPGA also outperforms PSO [30] and GA [29] in terms of a lower NMSE. Based on the results plotted in Figure 19b, the proposed ResNet obtains the lowest NMSE across a range of SNR regimes. The NMSE obtained by CRNN [17] is minor. Figure 20 displays the reflected channel estimation performance in UPA. Relying on the results shown in Figure 20a,b, the proposed MTRnet simultaneously obtains a lower NMSE compared with that achieved by CRNN [17] and CNN [15].

## 5. Conclusions

In this paper, we proposed a novel two-step channel estimation method for RIS-assisted mmWave systems. In the first step, the proposed MMPGA-LS-ResNet is introduced for cascaded channel estimation. The MMPGA-LS is capable of reducing the NMSE compared with some existing methods. Furthermore, the proposed ResNet, with its strong non-linear processing ability, further reduces the cascaded channel estimation NMSE. Based on the output of ResNet, the proposed MTRnet, integrating multi-task regression model and ResNet, can estimate multiple reflected channels simultaneously. Remarkably, the MTRnet has a lower number of optimization models compared with CRNN [17] and CNN [15]. Besides, the proposed MTRnet outperforms CRNN [17] and CNN [15] in terms of lower NMSE. The future work will focus on the active RIS-mmWace systems and optimization of neural networks.

## Figures and Tables

**Table 1:** Configuration of some primary layers in the proposed ResNet.

$\{{f^{-1}\}}_{i}$	Layers	Description
i=2,6,11,15	Convolution	64:3×3
i=3,7,12	Convolution	128:3×3
i=4,8,13	Convolution	256:3×3
i=5,10	Convolution	32:3×3
i=9,14	Cross-layers	-

**Table 2:** Configuration of network parameters in the five sub-tasks.

Sub-Tasks	Layers	Description
Sub-task I	hidden	*P* neurons
Sub-task II	hidden	*P* neurons
Sub-task III	hidden	2P neurons
Sub-task IV	hidden	*P* neurons
Sub-task V	hidden	2P neurons

**Table 3:** Computational complexity comparison of different methods.

Method	Complexity
MMPGA	$O(TM_{\mathrm{acti}}NL_{s}^{4}\left(Q^{2}+Q_{4}+Q_{4}L_{c}\right))$
PSO [30]	$O(2M_{\mathrm{acti}}TQNL_{s}^{4})$
GA [29]	$O(TM_{\mathrm{acti}}NL_{s}^{4}\left(Q^{2}+2Q\right))$
ResNet+MTRnet	$\sum_{l_{c}=1}^{L_{\mathrm{conv}}-1}\left(N_{x}N_{y}C_{\mathrm{in}}+1\right)C_{\mathrm{out}}+\sum_{l_{d}=1}^{L_{\mathrm{hidd}}-1}\left(D_{\mathrm{in}}+1\right)D_{\mathrm{out}}$
CRNN [17]	$\sum_{l_{c}=1}^{L_{\mathrm{CRNN},\mathrm{conv}}-1}\left(P+3\right)\left(N_{\mathrm{CRNN},x}N_{\mathrm{CRNN},y}C_{\mathrm{CRNN},\mathrm{in}}+1\right)C_{\mathrm{CRNN},\mathrm{out}}+$ld=1LCRNN,hidd-1(DCRNN,in+1)DCRNN,out
CNN [15]	$\sum_{l_{c}=1}^{L_{\mathrm{CNN},\mathrm{conv}}-1}\left(P+3\right)\left(N_{\mathrm{CNN},x}N_{\mathrm{CNN},y}C_{\mathrm{CNN},\mathrm{in}}+1\right)C_{\mathrm{CNN},\mathrm{out}}+$ld=1LCNN,hidd-1(DCNN,in+1)

## Data Availability

Data are contained within the article.
